# Vertical distribution of bacterial community diversity in the Greater Khingan Mountain permafrost region

**DOI:** 10.1002/ece3.9106

**Published:** 2022-07-11

**Authors:** Xin Li, Yuanquan Cui, Dalong Ma, Dandan Song, Lin Liu

**Affiliations:** ^1^ College of Geographical Sciences Harbin Normal University Harbin China; ^2^ Personnel department Harbin Normal University Harbin China

**Keywords:** bacterial diversity, high‐throughput sequencing, permafrost, vertical distribution

## Abstract

Soil microorganisms are crucial contributors to the function of permafrost ecosystems, as well as the regulation of biogeochemical cycles. However, little is known about the distribution patterns and drivers of high‐latitude permafrost microbial communities subject to climate change and human activities. In this study, the vertical distribution patterns of soil bacterial communities in the Greater Khingan Mountain permafrost region were systematically analyzed via Illumina Miseq high‐throughput sequencing. Bacterial diversity in the active layer was significantly higher than in the permafrost layer. Principal coordinate analysis (PCoA) indicated that the bacterial community structure in the active layer and the permafrost layer was completely separated. Permutational multivariate analysis of variance (PERMANOVA) detected statistically significant differentiation across the different depths. The relative abundance of the dominant phyla Chloroflexi (17.92%–52.79%) and Actinobacteria (6.34%–34.52%) was significantly higher in the permafrost layer than in the active layer, whereas that of Acidobacteria (4.98%–38.82%) exhibited the opposite trend, and the abundance of Proteobacteria (2.49%–22.51%) generally decreased with depth. More importantly, the abundance of bacteria linked to human infectious diseases was significantly higher in the permafrost layer according to Tax4Fun prediction analysis. Redundancy analysis (RDA) showed that ammonium nitrogen (NH_4_^+^‐N), total organic carbon (TOC), and total phosphorus (TP) were major factors affecting the bacterial community composition. Collectively, our findings provide insights into the soil bacterial vertical distribution patterns and major environmental drivers in high‐latitude permafrost regions, which is key to grasping the response of cold region ecosystem processes to global climate changes.

## Graphical abstract

Our study employed Illumina Miseq high‐throughput sequencing to characterize the vertical distribution patterns of soil microbial community structures and the key environmental driving factors in the active and permafrost layers of the Larix gmelinii forest located in the Greater Khingan Mountain permafrost region. Therefore, this study provides a scientific basis for accurately predicting and assessing the response of high‐latitude permafrost ecosystems to climate change.

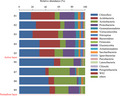

## 1 INTRODUCTION

Permafrost refers to all types of ice‐covered rock and soil that remain at temperatures below 0°C for more than 2 years, and these regions are among the most extreme environments on earth (Jansson & Taş, 2014). Permafrost is a crucial component of the cryosphere and plays a vital role in the global climate system (Heffernan et al., 2021). Climate warming is causing widespread permafrost degradation on a global scale, which is mainly manifested by increasing soil temperatures, shrinking permafrost areas, expanding thaw zones, and deepening the active layer thickness (Gao et al., 2021; Kim et al., 2016). The active layer is a transition layer that enables the exchange of water and heat between the permafrost layer and the atmosphere, and therefore constitutes a link between the atmosphere, the biosphere, and the hydrosphere (Steven et al., 2008). Changes in this transition layer can not only affect the hydrothermal properties of the soil itself and change the material balance within the soil but also have major implications for the ecological processes in entire cold regions (Helbig et al., 2016). The permafrost layer is transformed into the active layer through complex phase change processes, among which changes in temperature, moisture, and organic matter are highly likely to alter the biogeochemical cycles of key elements mediated by microorganisms under global warming conditions (Hultman et al., 2015). Therefore, microbial ecology associated with permafrost has recently garnered increasing attention in the context of global climate change.

Microorganisms in permafrost, particularly bacteria, can adjust to cryoenvironments and play a vital role in the decomposition and mineralization of soil organic matter, the circulation and transformation of soil nutrients (Fry et al., 2021). Permafrost areas in the northern hemisphere account for 24% of the land surface area (Graham et al., 2012). The organic carbon storage in this area accounts for approximately 50% of the global carbon storage, which is equivalent to the sum of the vegetation and atmospheric carbon storage (Gandois et al., 2019). The degradation of permafrost leads to the decomposition of long‐term sequestered organic carbon by microorganisms. In turn, this leads to the further release of greenhouse gases (CO_2_ and CH_4_), which are considered among the most important contributors to global climate change from terrestrial ecosystems (Yergeau et al., 2010). Soil profiles provide heterogeneous habitats for microorganisms, and bacterial distribution is strongly controlled by the bacterial dispersal ability and environmental conditions, which significantly affect bacterial diversity by driving species replacement or regulating differences in the richness of species (Gittel et al., 2014). The vertical distribution patterns of microbial communities in the unique permafrost soil remain relatively unexplored. Therefore, a deep understanding of the changes in microbial communities in different soil layers and the factors that form these communities is essential to predict the potential microbial processes and functions of permafrost ecosystem in climate change.

The Greater Khingan Mountain is the only cold‐temperate coniferous forest area in China, located on the southern edge of the high‐latitude permafrost zones of Eurasia, where the permafrost is fragile, thermally unstable, and vulnerable to climate and external environmental changes. The active layer experiences repeated freeze–thaw cycles and is subject to frequent disturbances by environmental factors, whereas the permafrost layer is an extreme environment with low moisture activity and extremely low nutrient conversion rates. Therefore, both of these environments are likely inhabited by different microbial flora (Mackelprang et al., 2011). Most current studies on soil microbial communities in permafrost regions have been conducted in the circumpolar Arctic, Siberia, Alaska, and the Qinghai‐Tibet Plateau (Aksenov et al., 2021; Singh et al., 2017; Tripathi et al., 2018; Wu et al., 2017), whereas much less attention has been given to the permafrost regions of the Greater Khingan Mountain, an area affected by both the effects of climate change and intense human activities, which limits our knowledge of the spatiotemporal variation trends and potential carbon feedback of permafrost under global warming conditions. The aim of this study was to (1) determine the vertical distribution patterns of soil bacterial community structures; (2) identify the key environmental factors driving the distribution of the bacterial community; and (3) reveal the soil bacterial predicted metabolic functions in the active and permafrost layers of the *Larix gmelinii* forest located in the Greater Khingan Mountain permafrost region based on Illumina Miseq high‐throughput sequencing. Therefore, this study provides a scientific basis for accurately predicting and assessing the response of high‐latitude permafrost ecosystems to climate change.

## 2 MATERIALS AND METHODS

### 2.1 Study area

The study area is located at Mohe Forest Ecosystem Research Station (53°17′–53°30′ N, 122°06′–122°27′ E) in Heilongjiang Province, China. The region exhibits a cold‐temperate continental monsoon climate, with mild and short summers followed by long cold winters. The average annual precipitation is 430 mm, and the average annual temperature is −4.9°C, with extreme minimum temperature as low as −49.5°C. The research area is widely covered with continuous permafrost, and the main soil type is dark brown forest soil (Liu et al., 2020). The active layer thickness is 0.7–3.0 m. The regional vegetation consists of cold‐temperate coniferous forests, which are largely dominated by *Larix gmelinii*. Other associated species include *Populus davidiana*, *Betula platyphylla*, and *Pinus sylvestris* var. *mongolica*.

### 2.2 Soil sampling

Three 20 m × 20 m sample plots were delimited in typical areas of the *Larix gmelinii* forest with consistent stand conditions. Next, 1.8‐m‐depth soil columns were drilled in each sample plot using a powered soil sampler (Drill bit was sterilized). These soil columns were divided into nine 20‐cm layers, of which B1‐B5 constituted the active layer and B6‐B9 were the permafrost layer. A total of 27 soil samples were obtained from the three columns. After removing stones, plant roots, and other debris, the samples were thoroughly mixed, placed in a sterilized ziplock bag, and taken back to the laboratory. A portion of the samples was naturally air‐dried and passed through a 2 mm sieve to characterize the physicochemical properties of the soil, and the remaining portion of the samples was stored at −80°C for gene sequencing.

### 2.3 Determination of soil physicochemical properties

Soil pH value was measured using a pH meter (PHS‐3E) at a 2.5:1 water‐to‐soil ratio. The soil samples were oven‐dried at 105°C to measure their soil water content (SWC). Soil total organic carbon (TOC) was determined using a total organic carbon analyzer (Multi N/C 3100, Germany). Soil nitrate nitrogen (NO_3_^−^‐N), ammonium nitrogen (NH_4_^+^‐N), total nitrogen (TN), and total phosphorus (TP) were measured with a continuous flow analyzer (SAN^++^, the Netherlands).

### 2.4 DNA extraction and sequencing

Total genomic DNA was extracted from the soil samples using the PowerSoil DNA Isolation Kit (MOBIO, USA), and DNA integrity was assessed via 0.8% agarose gel electrophoresis. 16S rRNA genes in V3‐V4 hypervariable regions were amplified with the forward primers (CCTACGGRRBGCA SCAGKVRVGAAT) and the reverse primers (GGACTACNVGGGTWTCTAATCC). Library quality was assessed using an Agilent 2100 Bioanalyzer system (Agilent Technologies, USA), after which it was quantified using a Qubit 2.0 Fluorometer (Thermo, USA). DNA libraries were multiplexed and sequencing was performed using a 2 × 300 paired‐end (PE) configuration in the MiSeq instrument by GENEWIZ, Inc. (Suzhou, China).

### 2.5 Data processing and statistical analyses

Paired‐end sequences were joined and processed using QIIME 2 (Hall & Beiko, 2018) and Mothur (v.1.45.3; Schloss et al., 2009). Removing chimeras and low‐quality reads using VSEARCH (v.2.6.0; Rognes et al., 2016) after quality control, which included sequences less than 50 bp, sequences with an average quality score of less than 20, inconsistent sequences after paired reads assembly, and sequences without the correct primer. The sequences were then assigned to operational taxonomic units (OTUs) using VSEARCH (v.2.6.0) at a 97% similarity level. The RDP classifier (Ribosomal Database Program) algorithm was applied through the SILVA_138 16S rRNA database to assign taxonomy to a species level and removed non‐bacteria, mitochondrial, and chloroplast OTUs. To reduce the impacts on differing read numbers across samples, the number of sequences of all samples was rarefied to the lowest read number using the R package *phyloseq* (rarify depth: 41709; McMurdie & Holmes, 2013). Applying a taxon filtering script provided by QIIME 2 was to separate the OTU tables of single microbial taxa, which were then analyzed the relative abundance of each specific taxa. The bacterial community composition was then described by the abundance of the sequences assigned to each taxon. The heatmaps of the bacterial relative abundance among different samples were created using R package *gplots* at the phylum and genus level classification (Warnes et al., 2020). Alpha diversity analysis (Good's coverage index, Chao1 index, ACE index, Simpson index, Shannon index, and Phylogenetic diversity index) and principal coordinate analysis (PCoA) based on Bray–Curtis distances were conducted using QIIME 2 and the R package *vegan* (Dixon, 2003). The diagram of shared and unique OTUs was generated with the R package *VennDiagram* (Chen & Boutros, 2011). Permutational multivariate analysis of variance (PERMANOVA) was performed to identify significant differences in bacterial community composition across the different depths (*adonis* in the R package *vegan*; Desgarennes et al., 2014). The functional contributions of the bacterial community in the soil samples were predicted based on the OTU using the Tax4Fun (v.0.3.1; Aßhauer et al., 2015). Differences in the physicochemical characteristics of soil samples obtained at different depths were identified via one‐way ANOVA using SPSS 20.0. The relationship between microbial communities and soil environmental parameters was assessed through redundancy analysis (RDA) using CANOCO 5.0 (Šmilauer & Lepš, 2014). The significance of the RDA correlations was determined by a Monte Carlo test, and the eigenvalues of the first two axes were used to reflect their important role. Raw sequences were deposited in the NCBI public database under the accession number of PRJNA818343.

## 3 RESULTS

### 3.1 Soil physicochemical properties at different depths

The soil water content (SWC) in the active layer (B1–B5) decreased with increasing depth and tended to increase in the permafrost layer (B6–B9). The SWC (40.90%) was significantly higher at B1 (0–20 cm) than other depths (*p* < .05; Table 1). The soil pH varied from 5.32 to 6.65 and was highest at B7 (120–140 cm). Total nitrogen (TN), nitrate nitrogen (NO_3_^−^‐N), and ammonium nitrogen (NH_4_^+^‐N) contents decreased with soil depth in the active layer and were markedly higher at B1 than at other depths (*p* < .05). Except for NO_3_^−^‐N (B6, 100–120 cm), the soil NH_4_^+^‐N and TN contents did not differ significantly in the permafrost layer (*p* > .05). The total organic carbon (TOC) and total phosphorus (TP) contents increased first, then decreased, and then increased with depth. These parameters were significantly higher at B2 (20–40 cm) compared to other depths (*p* < .05).

**TABLE 1:** Soil physical and chemical properties under different depths

	Sample	SWC (%)	pH	TOC (g/kg)	NH_4_^+^‐N (mg/kg)	NO_3_^−^‐N (mg/kg)	TN (g/kg)	TP (mg/kg)
Active layer	B1 (0–20 cm)	40.90 ± 5.57^a^	6.26 ± 0.19^ab^	37.56 ± 3.46^b^	15.34 ± 0.89^a^	5.75 ± 0.39^a^	2.55 ± 0.24^a^	993.59 ± 35.74^c^
	B2 (20–40 cm)	25.43 ± 3.38^b^	6.08 ± 0.28^b^	53.74 ± 2.59^a^	13.22 ± 1.74^b^	2.97 ± 0.27^b^	2.06 ± 0.18^b^	1658.06 ± 101.29^a^
	B3 (40–60 cm)	23.16 ± 1.84^b^	6.20 ± 0.32^ab^	41.35 ± 5.46^b^	12.56 ± 2.95^b^	2.55 ± 0.33^b^	1.72 ± 0.09^bc^	1362.23 ± 46.12^b^
	B4 (60–80 cm)	22.47 ± 2.14^b^	5.78 ± 0.25^bc^	26.04 ± 4.22^c^	11.65 ± 1.13^bc^	1.58 ± 0.25^c^	1.53 ± 0.11^c^	796.83 ± 85.71^c^
	B5 (80–100 cm)	17.52 ± 1.10^c^	5.32 ± 0.08^c^	20.01 ± 3.34^cd^	9.05 ± 1.30^c^	1.33 ± 0.36^cd^	1.49 ± 0.08^c^	840.33 ± 42.05^c^
Permafrost layer	B6 (100–120 cm)	19.62 ± 3.31^bc^	5.85 ± 0.42^bc^	17.27 ± 1.33^d^	11.59 ± 1.88^bc^	1.18 ± 0.09^d^	1.61 ± 0.15^c^	649.56 ± 77.47^cd^
	B7 (120–140 cm)	20.07 ± 2.90^bc^	6.65 ± 0.20^a^	14.04 ± 2.05^d^	10.17 ± 2.70^c^	2.11 ± 0.22^bc^	1.64 ± 0.21^bc^	514.05 ± 36.05^d^
	B8 (140–160 cm)	25.14 ± 5.49^b^	5.81 ± 0.15^bc^	13.05 ± 1.98^d^	11.27 ± 1.18^bc^	1.93 ± 0.31^bc^	1.50 ± 0.05^c^	580.71 ± 80.26^cd^
	B9 (160–180 cm)	25.25 ± 1.67^b^	6.24 ± 0.37^ab^	17.93 ± 0.78^cd^	10.30 ± 2.53^c^	1.56 ± 0.23^c^	1.62 ± 0.17^bc^	616.26 ± 51.08^cd^

*Note*: Different letters indicate significant difference among soil depths by one‐way ANOVA (LSD, *p* < .05). Data shown are mean values ± standard deviation (*n* = 3).

Abbreviations: NH_4_^+^‐N, ammonium nitrogen; NO_3_^−^‐N, nitrate nitrogen; SWC, soil water content; TOC, total organic carbon; TN, total nitrogen; TP, total phosphorus.

### 3.2 OTU statistics and bacterial community diversity

**FIGURE 1:**
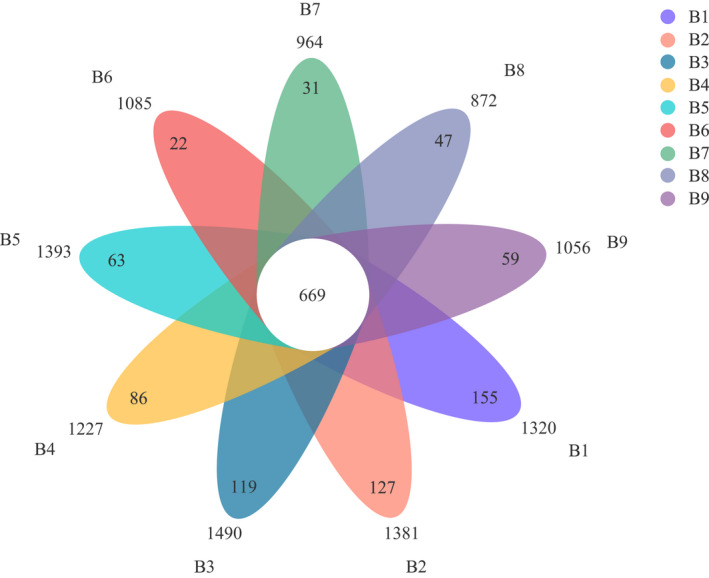
Venn diagram of soil bacterial community structure based on OTU level in different depths

The number of common and unique OTUs in the samples was visualized using Venn diagram (Figure 1). A total of 3097 soil bacterial OTUs were identified across all samples, of which 669 (21.60%) OTUs were shared, indicating that the OTU composition of the soil varied depending on depth. The highest number of specific bacterial OTUs was observed at B1 (155), and the lowest was observed at B6 (22). The number of specific OTUs decreased first and then increased with soil depth. As indicated in Table 2, the sample Good's coverage index was >98.49%, meaning that the sequencing depth was sufficient to assess the bacterial community structure and diversity. Both the ACE (1966.77) and Chao1 (1911.04) indices peaked at B3 (40–60 cm). These values increased first, then decreased, and then increased with soil depth. The highest Shannon (5.73) and PD (138.27) indices were observed at B1, which were significantly higher in the active layer (B1–B5) than in the permafrost layer (B6–B9; *p* < .05), whereas the Simpson index exhibited the opposite trend.

**TABLE 2:** Soil microbial diversity index of different depths

	Sample	ACE	Chao1	Shannon	Simpson	PD	Good's coverage (%)
Active layer	B1 (0–20 cm)	1663.77 ± 72.29^b^	1636.45 ± 110.04^b^	5.73 ± 0.15^a^	0.008 ± 0.001^b^	138.27 ± 17.29^a^	98.49 ± 0.14^a^
	B2 (20–40 cm)	1932.26 ± 147.95^a^	1825.10 ± 169.23^ab^	5.58 ± 0.25^a^	0.009 ± 0.001^b^	129.06 ± 8.40^a^	99.09 ± 0.45^a^
	B3 (40–60 cm)	1966.77 ± 160.56^a^	1911.04 ± 117.50^a^	5.69 ± 0.33^a^	0.011 ± 0.003^b^	122.91 ± 7.15^a^	98.78 ± 0.16^a^
	B4 (60–80 cm)	1550.60 ± 121.69^b^	1594.64 ± 98.79^b^	5.47 ± 0.08^a^	0.012 ± 0.002^b^	110.33 ± 10.38^a^	99.15 ± 0.31^a^
	B5 (80–100 cm)	1769.86 ± 193.18^ab^	1721.52 ± 137.83^ab^	5.52 ± 0.27^a^	0.013 ± 0.002^b^	123.22 ± 11.71^a^	98.65 ± 0.28^a^
Permafrost layer	B6 (100–120 cm)	1404.67 ± 63.22^c^	1339.59 ± 118.75^c^	5.04 ± 0.35^b^	0.019 ± 0.003^a^	88.47 ± 8.25^b^	99.21 ± 0.09^a^
	B7 (120–140 cm)	1235.92 ± 79.17^d^	1201.13 ± 65.60^d^	4.58 ± 0.09^b^	0.027 ± 0.006^a^	73.09 ± 14.33^b^	99.89 ± 0.06^a^
	B8 (140–160 cm)	1036.61 ± 127.92^e^	1077.23 ± 113.68^e^	4.61 ± 0.14^b^	0.024 ± 0.005^a^	71.76 ± 12.73^b^	99.82 ± 0.13^a^
	B9 (160–180 cm)	1347.03 ± 104.11^c^	1361.03 ± 135.80^c^	5.09 ± 0.17^b^	0.018 ± 0.003^a^	82.78 ± 4.81^b^	99.62 ± 0.04^a^

*Note*: Different letters indicate significant difference among soil depths by one‐way ANOVA (LSD, *p* < .05). Data shown are mean values ± standard deviation (*n* = 3).

### 3.3 Soil bacterial community structure and potential metabolic pathways

**FIGURE 2:**
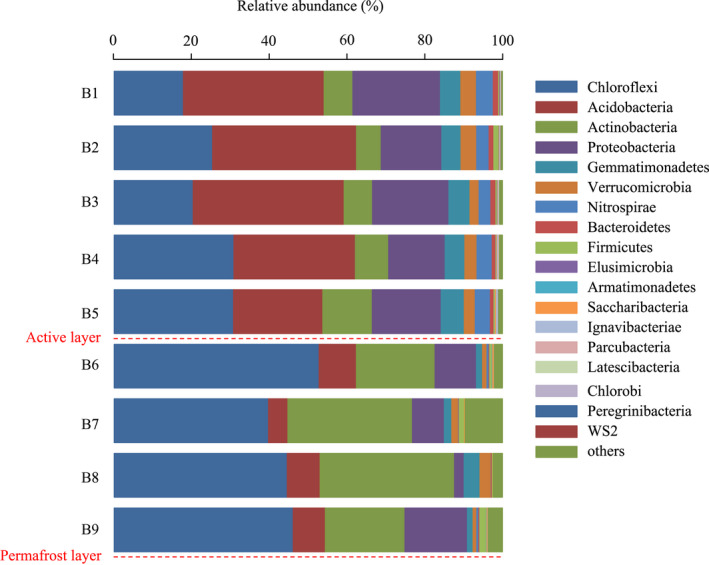
Characteristics of bacterial community structure at the level of phylum in different depths

The soil samples at the nine depths were dominated by Chloroflexi (17.92%–52.79%), Acidobacteria (4.98%–38.82%), Actinobacteria (6.34%–34.52%), and Proteobacteria (2.49%–22.51%) at the phylum level. However, their relative abundance varied greatly between depths (Figure 2). The relative abundance of Chloroflexi was highest at B6, whereas Actinobacteria peaked at B8 (140–160 cm). Their abundance was significantly higher in the permafrost layer (B6‐B9) than in the active layer (B1‐B5; *p* < .05). The relative abundance of Acidobacteria was highest at B3 and was markedly higher in the active layer than in the permafrost layer (*p* < .05). The relative abundance of Proteobacteria peaked at B1 and decreased with soil depth, but increased significantly at B9 (160–180 cm; *p* < .05). Moreover, the abundance of Gemmatimonadetes (1.46%–5.94%), Verrucomicrobia (0.96%–4.04%), and Nitrospirae (0.07%–4.31%) was higher in the active layer than in the permafrost layer.

**FIGURE 3:**
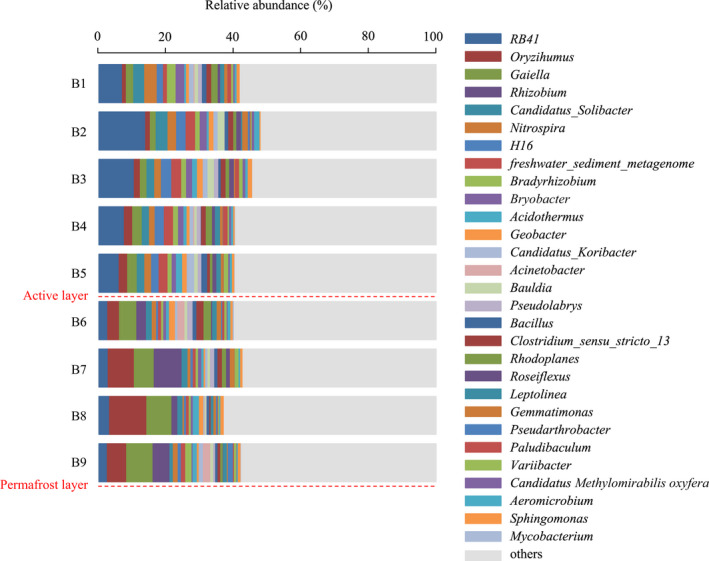
Characteristics of bacterial community structure at the level of genus in different depths

At the genus level, the top 10 bacteria based on relative abundance were *RB41* (2.43%–13.95%), *Oryzihumus* (1.15%–11.04%), *Gaiella* (1.69%–7.82%), *Rhizobium* (0%–8.25%), *Candidatus_Solibacter* (0.98%–3.52%), *Nitrospira* (0.41%–3.72%), *H16* (0.20%–3.05%), *freshwater_sediment_metagenome* (0.33%–2.94%), *Bradyrhizobium* (0.58%–2.55%), and *Bryobacter* (0.35%–2.41%; Figure 3). The relative abundance of *RB41*, *Candidatus_Solibacter*, *Nitrospira*, *H16*, *freshwater_sediment_metagenome*, *Bradyrhizobium*, and *Bryobacter* in the active layer was significantly higher than that in the permafrost layer, whereas the abundance of *Oryzihumus* and *Gaiella* exhibited the opposite trend (*p* < .05). Additionally, *Rhizobium* occurred only in the permafrost layer, and the relative abundance was markedly higher at B7 and B9 than at B6 and B8 (*p* < .05).

**FIGURE 4:**
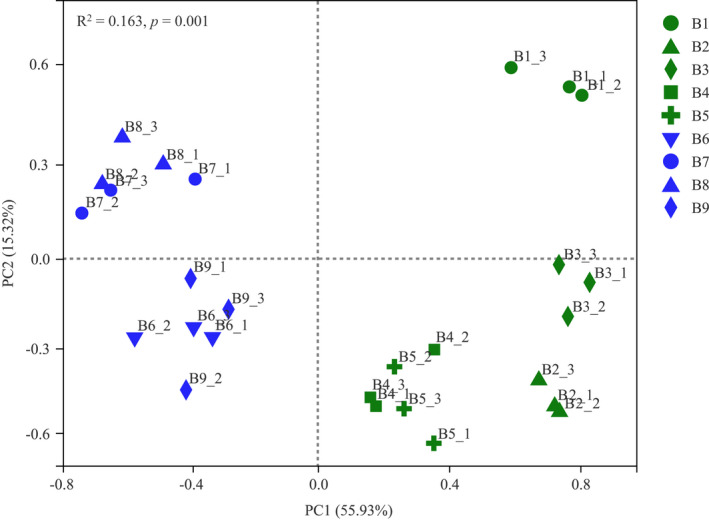
Principal coordinate analysis (PCoA) of soil bacteria in different depths. Green represents active layer samples, blue represents permafrost layer samples. The results of the PERMANOVA were indicated on the left corner

PCoA (calculated on Bray–Curtis) indicated that the cumulative contribution rate of PC1 and PC2 was 71.25%, of which the bacterial community structure in the active layer (B1‐B5) and the permafrost layer (B6‐B9) was completely separated on the PC1 axis, forming two distinct clusters (Figure 4). In the active layer, the bacterial community of B1 was clearly distinguishable from B2 to B5. The permafrost samples were divided into two groups, B7‐B8 samples occurred in one cluster, whereas B6 was relatively close to B9. PERMANOVA analysis indicated that there were significant differences of bacterial community composition across the different depths (*R* ^2^ = 0.163, *p* = .001).

**FIGURE 5:**
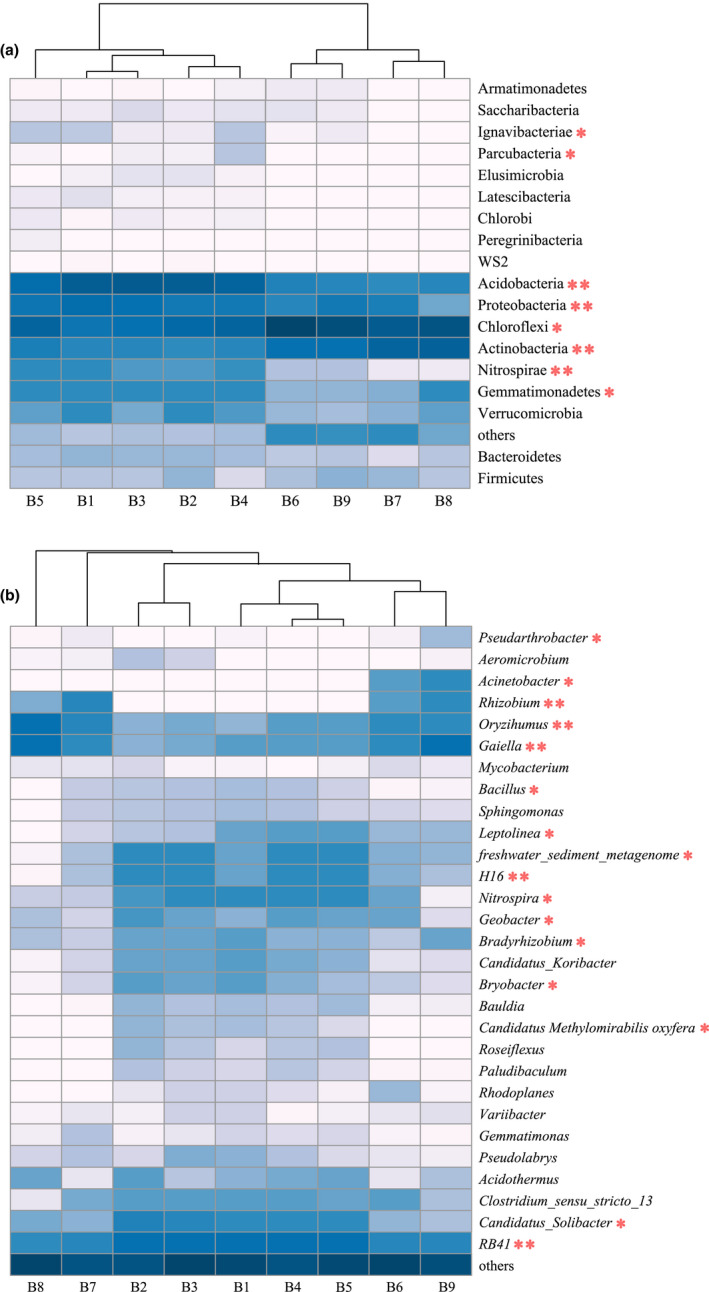
Heatmaps of bacterial composition differences at the phylum (a) and genus (b) level. Statistical significance in bacterial relative abundance in different depths was determined by one‐way ANOVA. * means significant at *p* < .05, ** means significant at *p* < .01

The relative abundance of Gemmatimonadetes, Nitrospirae, Actinobacteria, Chloroflexi, Proteobacteria, Acidobacteria, Parcubacteria, and Ignavibacteriae exhibited significant depth‐dependent differences at the phylum level (*p* < .05; Figure 5a). At the genus level, the relative abundance of *RB41*, *H16*, *Bryobacter*, *Bradyrhizobium*, *Nitrospira*, *Gaiella*, *freshwater_sediment_metagenome*, *Leptolinea*, *Geobacter*, *Candidatus_Solibacter*, *Candidatus Methylomirabilis oxyfera*, *Oryzihumus*, *Rhizobium*, *Bacillus*, *Acinetobacter*, and *Pseudarthrobacter* varied markedly at different soil depths (*p* < .05; Figure 5b).

Functional annotation of six types of Level 1 functional groups and 40 types of Level 2 functional groups was conducted using Tax4Fun. Table 3 summarizes the taxa with relative abundance >1%. ANOVA analyses of the Level 2 metabolic pathways indicated that the abundance of energy metabolism, nucleotide metabolism, and membrane transport was significantly higher in the active layer, whereas that of xenobiotic biodegradation and metabolism was higher in the permafrost layer. Additionally, the abundance of bacteria associated with human infectious diseases was significantly higher in the permafrost layer (except B9) than in the active layer (*p* < .05).

**TABLE 3:** Bacterial community functions predicted by Tax4Fun (level 2)

Pathway level 1	Pathway level 2	Active layer					Permafrost layer			
		B1	B2	B3	B4	B5	B6	B7	B8	B9
Metabolism	Carbohydrate metabolism	12.59 ± 0.06^a^	13.23 ± 0.21^a^	12.88 ± 0.09^a^	12.70 ± 0.04^a^	12.90 ± 0.13^a^	12.67 ± 0.11^a^	12.72 ± 0.09^a^	12.50 ± 0.07^a^	12.62 ± 0.15^a^
	Amino acid metabolism	11.85 ± 0.13^a^	12.10 ± 0.06^a^	11.75 ± 0.23^a^	12.02 ± 0.10^a^	11.66 ± 0.08^a^	12.18 ± 0.18^a^	11.60 ± 0.07^a^	11.84 ± 0.05^a^	11.50 ± 0.22^a^
	Energy metabolism	7.66 ± 0.24^a^	7.88 ± 0.18^a^	7.46 ± 0.06^a^	7.62 ± 0.05^a^	7.47 ± 0.11^a^	6.50 ± 0.14^b^	6.33 ± 0.06^b^	6.21 ± 0.11^b^	6.31 ± 0.08^b^
	Metabolism of cofactors and vitamins	6.80 ± 0.09^a^	6.85 ± 0.25^a^	6.76 ± 0.07^a^	6.85 ± 0.04^a^	6.68 ± 0.01^a^	6.75 ± 0.03^a^	6.71 ± 0.07^a^	6.92 ± 0.25^a^	6.77 ± 0.13^a^
	Nucleotide metabolism	5.47 ± 0.02^a^	5.24 ± 0.06^a^	5.30 ± 0.11^a^	5.17 ± 0.16^a^	5.32 ± 0.09^a^	4.72 ± 0.02^b^	4.53 ± 0.05^b^	4.24 ± 0.17^b^	4.46 ± 0.06^b^
	Xenobiotics biodegradation and metabolism	4.21 ± 0.03^b^	4.03 ± 0.02^b^	4.14 ± 0.06^b^	4.13 ± 0.10^b^	4.16 ± 0.09^b^	4.60 ± 0.17^a^	4.49 ± 0.23^a^	4.76 ± 0.05^a^	4.72 ± 0.06^a^
	Lipid metabolism	3.61 ± 0.12^a^	3.63 ± 0.06^a^	3.58 ± 0.04^a^	3.50 ± 0.13^a^	3.65 ± 0.21^a^	3.53 ± 0.09^a^	3.55 ± 0.04^a^	3.58 ± 0.07^a^	3.70 ± 0.06^a^
	Metabolism of terpenoids and polyketides	2.60 ± 0.06^ab^	2.51 ± 0.01^b^	2.79 ± 0.02^a^	2.70 ± 0.02^ab^	2.64 ± 0.05^ab^	2.79 ± 0.11^a^	2.86 ± 0.04^a^	2.90 ± 0.10^a^	2.68 ± 0.08^ab^
	Glycan biosynthesis and metabolism	2.08 ± 0.03^a^	2.16 ± 0.02^a^	2.12 ± 0.04^a^	2.14 ± 0.12^a^	2.31 ± 0.10^a^	2.29 ± 0.06^a^	2.16 ± 0.04^a^	2.21 ± 0.03^a^	2.22 ± 0.07^a^
Environmental information processing	Membrane transport	14.22 ± 0.22^a^	13.92 ± 0.09^a^	13.68 ± 0.04^a^	13.75 ± 0.10^a^	13.85 ± 0.15^a^	12.53 ± 0.05^b^	12.30 ± 0.07^b^	12.03 ± 0.09^b^	12.13 ± 0.06^b^
	Signal transduction	7.53 ± 0.04^ab^	7.65 ± 0.12^a^	7.77 ± 0.03^a^	7.64 ± 0.31^a^	7.24 ± 0.14^b^	7.68 ± 0.08^a^	7.82 ± 0.02^a^	7.48 ± 0.07^ab^	7.04 ± 0.16^b^
Genetic information processing	Translation	4.17 ± 0.05^ab^	4.11 ± 0.08^b^	4.24 ± 0.06^a^	4.19 ± 0.01^ab^	4.33 ± 0.03^a^	4.42 ± 0.13^a^	4.29 ± 0.17^a^	4.32 ± 0.05^a^	4.09 ± 0.05^b^
	Replication and repair	3.75 ± 0.02^a^	3.71 ± 0.06^a^	3.92 ± 0.11^a^	3.89 ± 0.17^a^	4.01 ± 0.08^a^	3.70 ± 0.14^a^	3.71 ± 0.08^a^	3.80 ± 0.06^a^	3.67 ± 0.19^a^
	Folding, sorting and degradation	2.11 ± 0.03^a^	2.14 ± 0.04^a^	2.17 ± 0.06^a^	2.02 ± 0.02^ab^	2.06 ± 0.13^ab^	2.19 ± 0.03^a^	2.21 ± 0.07^a^	2.13 ± 0.27^a^	1.99 ± 0.18^b^
Cellular processes	Cell motility	2.43 ± 0.05^a^	2.47 ± 0.15^a^	2.49 ± 0.01^a^	2.44 ± 0.04^a^	2.51 ± 0.04^a^	2.29 ± 0.01^a^	2.19 ± 0.12^b^	2.27 ± 0.06^a^	2.21 ± 0.05^ab^
	Cell growth and death	1.70 ± 0.11^a^	1.88 ± 0.06^a^	1.82 ± 0.13^a^	1.66 ± 0.03^a^	1.80 ± 0.06^a^	1.90 ± 0.04^a^	1.66 ± 0.02^a^	1.65 ± 0.07^a^	1.84 ± 0.06^a^
Human diseases	Infectious disease: bacterial	1.08 ± 0.09^b^	1.20 ± 0.04^b^	1.01 ± 0.05^b^	1.08 ± 0.11^b^	1.22 ± 0.10^b^	1.81 ± 0.07^a^	1.73 ± 0.09^a^	1.62 ± 0.11^a^	1.15 ± 0.06^b^

*Note*: Different letters indicate significant difference among soil depths by one‐way ANOVA (LSD, *p* < .05). Data shown are mean values ± standard deviation (*n* = 3).

### 3.4 Effect of soil physicochemical properties on the bacterial community structure

**FIGURE 6:**
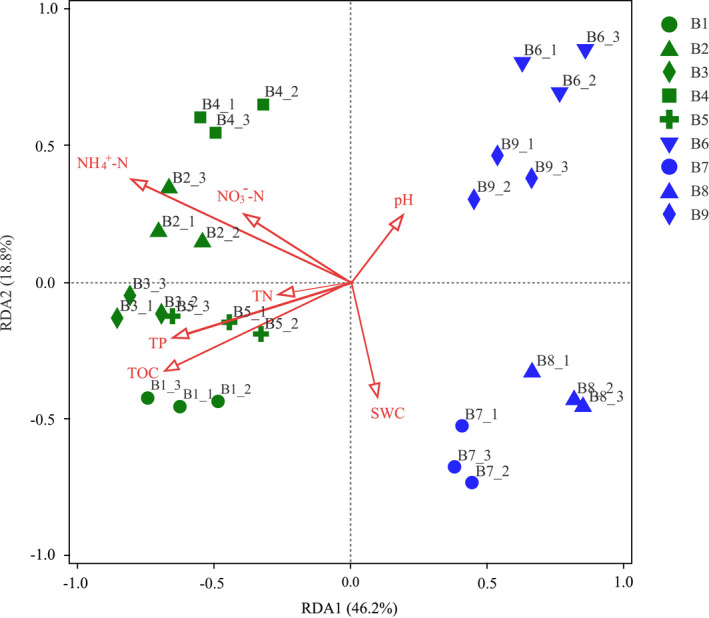
Redundancy analysis (RDA) of bacterial community structure and environmental factors in different depths. Green represents active layer samples, blue represents permafrost layer samples. Abbreviations: SWC, soil water content; TOC, total organic carbon; NH_4_^+^‐N, ammonium nitrogen; NO_3_^−^‐N, nitrate nitrogen; TN, total nitrogen; TP, total phosphorus

RDA indicated that the two axes explained 46.2% and 18.8% of the changes in the bacterial community structure, respectively (Figure 6). Furthermore, the effect of environmental factors on the bacterial community exhibited the following decreasing order: NH_4_^+^‐N > TOC > TP > NO_3_^−^‐N > SWC > TN > pH. Among these factors, the distribution of the bacterial community was obviously affected by NH_4_^+^‐N (*F* = 6.1, *p* = .004), TOC (*F* = 5.0, *p* = .016), and TP (*F* = 4.6, *p* = .016), whereas NO_3_^−^‐N, TN, SWC and pH had a lower influence on community structure (*p* > .05).

## 4 DISCUSSION

In our study, the bacterial community diversity varied significantly between the active layer and permafrost layer. Studies in the Canadian High Arctic (Jansson & Taş, 2014), Alaskan Arctic (Ji et al., 2020), and Siberian (Belov et al., 2020) permafrost confirmed that the soil microbial diversity was highest in the active layer and decreased with depth in the permafrost layer. Compared with the active layer, the permafrost layer exhibits several unique characteristics, including low temperatures, low oxygen levels, and low water availability. As a powerful ecological filter that limits microbial colonization, the permafrost layer reduces microbial diversity (Tripathi et al., 2018). The permafrost layer remains frozen all year round with a small amount of unfrozen water, thereby limiting the bacterial metabolic activity and completely changing the microbial niche. Interestingly, the diversity and community composition of soil bacteria show stratum specificity. Soil depth can alter soil nutrient availability, which affects the microbial community by regulating the physiological activities of microorganisms. Litter and root exudates control soil microbial community composition and diversity by providing substrates and nutrients (Millard & Singh, 2010). In the high‐latitude permafrost region of the Greater Khingan Mountain, there was an extremely high amount of litter input on the soil surface derived from the *Larix gmelinii* forest, and plant roots were concentrated at 0–80 cm. The soil nutrient increased and sustained microbial growth in the active layer, whereas microbial abundance was suppressed by low‐quality substrates in the permafrost layer (Aksenov et al., 2021).

In this study, significant spatial variations of soil microbial community composition were observed, and the main bacterial phyla in the Greater Khingan Mountain permafrost region were Chloroflexi, Acidobacteria, Actinobacteria, and Proteobacteria. This result was consistent with previous studies in the Antarctic, Arctic, and Qinghai‐Tibetan Plateau permafrost soils (Tytgat et al., 2016; Wilhelm et al., 2011; Wu et al., 2017). Chloroflexi preferentially grows in low nutrient and anaerobic environments, as confirmed by studies in the Greenland and Svalbard permafrost (Ganzert et al., 2014; Xue et al., 2020). Our findings indicated that the relative abundance of Chloroflexi was highest at the transition layer (100–120 cm, above the permafrost interface) and significantly higher in the permafrost layer than in the active layer, which was probably due to the ability of these bacteria to resist low temperatures and limited nutrient availability. Acidobacteria and Proteobacteria play important roles in material metabolism and organic matter decomposition. Due to their preference for nutrient‐rich environments, the higher abundances of Acidobacteria and Proteobacteria in the active layer may be due to an increase in litter and root exudate (Naumova et al., 2021). Consistent with this, other studies found that higher levels of TOC and TN increase the abundance of Acidobacteria and Proteobacteria in different soil depths (Eichorst et al., 2018; Frey et al., 2021). Furthermore, our findings indicated that Actinobacteria was predominant in the permafrost layer, and the dominant genera in the permafrost soil (*Oryzihumus* and *Gaiella*) also belonged to the Actinobacteria. By efficiently hydrolyzing complex organic compounds such as starch, cellulose, and xylan, Actinobacteria can maintain metabolic activity and cope with nutrient limitation at low temperatures (Chapman et al., 2017). More importantly, our study found that the lowest TOC content and the highest Actinobacteria abundance occurred at 140–160 cm. Similarly, Fierer et al. (2003) also reported that Actinobacteria was adapted to low‐carbon environments. In summary, there was a shift in the dominant soil bacteria from Acidobacteria and Proteobacteria dominance in the active layer to Chloroflexi and Actinobacteria dominance in the permafrost layer. Zhang et al. (2017) used the increase in the Actinobacteria to Proteobacteria ratio as a signal of permafrost degradation on the Qinghai‐Tibet Plateau, suggesting the necessity of more attention to the possibility of these bacterial taxa as indicator bacteria of potential environmental changes in the future. Our findings also indicated that *Rhizobium* occurred only in the permafrost layer, and Singh et al. (2017) reached the same conclusions in their study of the Arctic permafrost. However, the role of this bacterial genus in the permafrost soil microbiome remains unclear. Long‐term freezing constraints may be a more crucial determinant of microbial community composition than soil depth itself in permafrost soils, and once these constraints on permafrost thawing are relieved, microorganisms in the active layer seem to migrate and rapid metabolic responses can promote the decomposition of soil organic matter, which may increase carbon losses in the short term.

Group metabolism was the most commonly identified (58.36%–62.59%) function in the soil samples, followed by environmental information processing (19.81%–21.93%). Our study also identified differences in Level 2 metabolic pathways (energy metabolism, nucleotide metabolism, membrane transport, xenobiotics biodegradation, and metabolism) between the active layer and permafrost layer. Notably, the abundance of bacteria linked to human infectious diseases was significantly higher at 100–160 cm (B6‐B8, permafrost layer) than at other depths. Recent studies have demonstrated that increased access to the Arctic, as well as the potential thawing of materials that had long remained frozen, are increasing the likelihood of the liberation of infectious diseases from the permafrost layer (Waits et al., 2018). Stella et al. (2020) also hypothesized that the release of *Bacillus anthracis* from the active layer contributed to a recent anthrax outbreak in Siberia. As permafrost melts and the active layer deepens due to climate warming, it is critical to understand the structure and function of microbial communities in permafrost soil and its human health implications. It is necessary to study metagenomic sequencing for accurately assessing the potential impact of permafrost microbial community functions on human health in the future.

Changes in physicochemical properties lead to variations in microbial communities with increasing soil depth (Ade et al., 2018). In this study, RDA demonstrated that soil NH_4_^+^‐N, TOC, and TP were the key drivers of soil microbial communities across all soil samples. Fierer et al. (2012) also found that soil NH_4_^+^‐N content was significantly correlated with bacterial community abundance and diversity. Moreover, NH_4_^+^‐N was the predominant form of inorganic nitrogen in boreal forest soil and was mainly present in an adsorbed state (Kothawala & Moore, 2009). In this study, soil NH_4_^+^‐N content decreased with depth in the active layer due to the freeze–thaw process, which damaged the soil aggregate structure and thus affected the nitrogen attachment capacity, reducing the soil nitrogen fixation effectiveness (Nagano et al., 2018). Differences in organic carbon availability at different soil depths may partly be responsible for variations in bacterial communities between active layers and permafrost layers (Deng et al., 2015). In turn, this results in physiological adaptations of bacteria in the permafrost layer, which enables them to use more complex organic carbon forms (Eilers et al., 2012). Because of nonlinear relationships between microbial communities and ecosystem properties and cascading influences of changes in these properties, the decrease in microbial community stability with the deepening of the active layer due to intensified permafrost thawing may potentially cause abrupt shifts in ecosystem states (Monteux et al., 2018). Especially, reduced stability of the microbial community in high‐latitude permafrost regions may cause increased organic carbon decomposition, which could induce positive warming feedbacks.

## 5 CONCLUSION

This study characterized the vertical distribution patterns of microbial communities in the Greater Khingan Mountain permafrost soils. Our results demonstrated that the soil microbial community diversity and composition exhibited significant spatial variations, and the changes were from Acidobacteria and Proteobacteria dominance in the active layer to Chloroflexi and Actinobacteria dominance in the permafrost layer. There was a strongly pronounced preference of *Oryzihumus*, *Rhizobium* and *Gaiella* for deeper permafrost layer. In contrast, *RB41*, *Candidatus_Solibacter*, *Nitrospira*, *H16*, *freshwater_sediment_metagenome*, *Bradyrhizobium*, and *Bryobacter* mostly present in the active layer. RDA provided further evidence that soil NH_4_^+^‐N, TOC, and TP predominantly explained the variability of soil bacterial community structures. Therefore, the present study provides important ecological insights into permafrost microbial communities and their drivers, which will be helpful in predicting their response to changes in high‐latitude permafrost ecosystems.

## AUTHOR CONTRIBUTIONS

**Xin Li:** Conceptualization (equal); data curation (equal); investigation (equal); writing – original draft (equal); writing – review and editing (equal). **Yuanquan Cui:** Conceptualization (equal); data curation (equal); software (equal); writing – review and editing (equal). **Dalong Ma:** Conceptualization (equal); data curation (equal); methodology (equal); writing – review and editing (equal). **Dandan Song:** Data curation (equal); methodology (equal); writing – review and editing (equal). **Lin Liu:** Data curation (equal); investigation (equal); software (equal); writing – original draft (equal).

## CONFLICT OF INTEREST

The authors declare that they have no conflict of interest.

## Data Availability

Raw sequences were deposited in the NCBI public database under the accession number of PRJNA818343.
